# Case series: MRI features in cerebral malaria

**DOI:** 10.4103/0971-3026.41832

**Published:** 2008-08

**Authors:** Sarmistha Gupta, Kailash Patel

**Affiliations:** Department of Radiodiagnosis, Bhopal Memorial Hospital and Research Centre, Bhopal - 462 038, MP, India

**Keywords:** Cerebral malaria, infarct, MRI

Malaria is an important tropical disease. Cerebral involvement is the most important complication in *Plasmodium falciparum* (*P. falciparum*) infection, with a mortality rate of up to 50%.[1] Imaging findings underestimate the extent of cerebral involvement in malaria, a normal CT brain being seen in many patients with cerebral involvement.

We would like to report the MRI findings in two patients with cerebral malaria. One patient had a hemorrhagic venous infarct in the left frontoparietal region and the other patient had signal intensity changes in the left parietal and insular cortex, similar to that seen in encephalitis.

## Case 1

A 21-year-old man presented with a history of fever, chills, and rigors for 12 days, along with generalized tonic-clonic seizures, occurring 2-3 times per day, for 5 days. He also had a history of loss of consciousness 2-3 times a day, each episode lasting for 2-3 min. He complained of loss of power in the right upper limb for 4 days. He had had 4-5 episodes of projectile vomiting during the entire duration of the illness. CNS examination revealed that he was conscious and oriented. There was no neck rigidity. Power in the right upper limb was 4/5. His sensory and motor systems were normal; there was no cranial nerve deficit. The other systemic examinations were normal. Biochemical and hematological investigations were normal. Peripheral smear for malarial parasite, done elsewhere, was positive and the patient was being treated with antimalarial drugs.

**Figure 1 (A, B):**
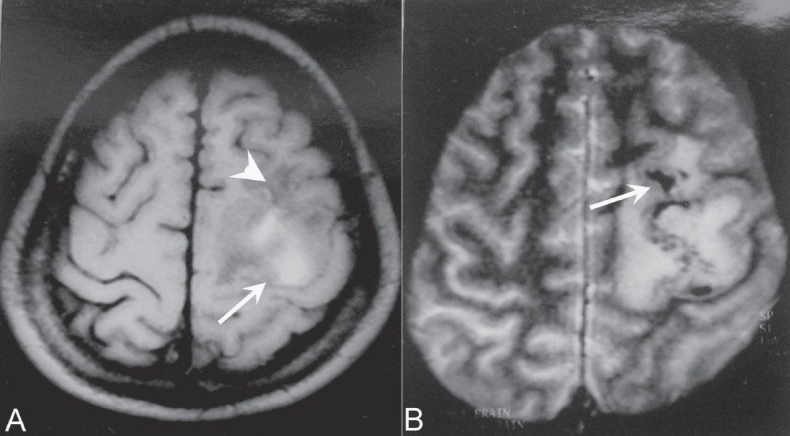
Axial T1W image demonstrates a hyperintense signal (arrow) in the high left frontoparietal region with surrounding hypointensity (arrowhead). The gradient image (B) shows blooming (arrow) within the T1 hyperintense area

MRI of the brain revealed an irregular lesion, hyperintense on T1W images [Figure 1A], with mixed hyper- and hypointense areas on the T2W images in the left high posterior frontoparietal region, which bloomed on gradient-echo images [Figure 1B]. Perilesional edema in the subcortical white matter with some involvement of the overlying gray matter was seen, suggesting the possibility of a hemorrhagic infarct. There was no evidence of venous thrombosis.

Two days later, the patient complained of pain and swelling in the left lower limb. Venous Doppler of the limb showed deep vein thrombosis. The patient was treated aggressively with antithrombotics, antibiotics, antipyretics, and anti-inflammatory drugs along with antacids. The patient had already completed a course of antimalarial drugs. He improved clinically. Follow-up CT scan of the brain done 1 month later showed a resolving infarct in the left frontoparietal region.

## Case 2

A 52-year-old patient with status epilepticus was brought for MRI of the brain. She had a history of fever with chills and rigors for the last 12 days. Her liver function tests and renal function tests were deranged and the peripheral smear was positive for malarial parasites. She was given antimalarial therapy and other supportive treatment, after which her renal and liver parameters improved. However, after 5-6 days, she developed right focal seizures followed by left focal seizures. The seizures responded initially to anticonvulsants but later became refractory to treatment and the patient went into status epilepticus. She had no other abnormality on examination. No other biochemical or hematological abnormality was found.

**Figure 2(A-C):**
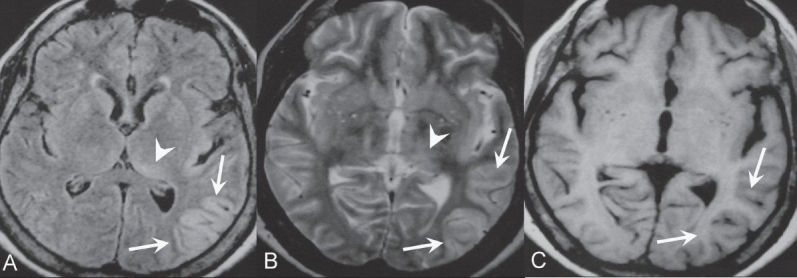
Axial FLAIR (A) and T2W (B) images demonstrate gyriform hyperintensity in the left parietal and insular cortex (arrow), which appears hypointense (arrows) on the axial T1W image (C). Note the ill-defined hyperintensity in the left thalamus (arrowhead in A and B)

Plain and post-contrast CT revealed no abnormality. MRI of the brain showed hyperintensity in the left thalamus and gyriform hyperintensity in the left parietal and insular cortex on FLAIR (fluid-attenuated inversion recovery) [Figure 2A] and T2W [Figure 2B] images. The T1W images showed corresponding hypointense areas [Figure 2C]. No abnormal enhancement was seen. The signal characteristics and distribution suggested encephalitis.

The patient gradually improved with treatment and was normal clinically and radiologically at her last follow-up.

## Discussion

Cerebral malaria is a life-threatening complication of *P. falciparum* infection. The infection is acquired after being bitten by an infected female Anopheles mosquito and is a multisystem disease, with hepatic dysfunction, thrombocytopenia, and coagulopathies. Neurological manifestations occur in 2% of cases of malaria. Cerebral damage in malaria is due to vascular sequestration of parasitized erythrocytes. A functional blockade of the microcirculation is the main pathogenetic mechanism and therefore diffuse bihemispheric dysfunction is the most common manifestation of cerebral involvement. Patients become drowsy and disoriented; hallucinations are common. As the disease process advances, the level of consciousness deteriorates and the patient becomes comatose.[2]

Cardoliani *et al.*[3] reported MRI findings in three cases of cerebral malaria. A cortical infarct was seen in one case, with hyperintense areas in the white matter on T2W and FLAIR images in the other two cases. The hyperintense white matter areas were either diffuse (which was attributed to white matter edema) or focal (probably due to gliosis).[3] In a prospective study of 20 patients with cerebral malaria, MRI showed that the brain volume during acute cerebral malaria was slightly more than during the convalescent phase of the disease.[4] This difference was attributed to an increase in the volume of intracerebral blood caused by sequestration of parasitized erythrocytes and compensatory vasodilatation, rather than by edema.[4] One patient even had herniation through the foramen magnum.

Hemorrhagic or bland infarcts in the basal ganglia, thalamus, or cerebellum are known to occur. An isolated pontine infarct[1] and a hemorrhagic infarct in the parietooccipital lobe have been reported as well.[5]

Koch *et al.*[6] reported a case of acute symptomatic psychosis occurring 2 weeks after successful therapy of a *P. falciparum* infection. MRI revealed multiple hyperintense lesions on the T2W images. They attributed these to an immune-mediated complication (acute disseminated encephalomyelitis, ADEM) rather than a direct result of the infection.[6]

The hyperintensities in the left parietal and insular cortex and the left thalamus, on the T2W and FLAIR images, which were seen in our second case, are most likely due to the edema of acute encephalitis. This appearance has not been previously reported in cerebral malaria to the best of our knowledge. A hemorrhagic infarct, as seen in our first case, has been reported only once before in cerebral malaria.[5] We report these two cases due to their unusual MRI appearances.

## References

[1] Kampfl AW, Birbaner GG, Pfausler BE, Haring HP, Schmutzhard E (1993). Isolated pontine lesion in algid cerebral malaria: Clinical features, management and magnetic resonance imaging findings. Am J Trop Med Hyg.

[2] Khadilkar SV, Sorabjee JS (1996). Cerebral malaria. Bombay Hosp Jr.

[3] Cordoliani YS, Sarrazin JL, Felten D, Caumes E, Leveque C, Fisch A (1998). MR of cerebral malaria. AJNR Am J Neuroradiol.

[4] Looareesuwan S, Wilairatana P, Krisha S, Kendall B, Vannaphan S, Viravan C (1995). Magnetic resonance imaging of the brain in patients with cerebral malaria. Clin Infect Dis.

[5] Millan JM, Sen Millan JM, Munoz M, Navas E, Lopez-Velez R (1993). CNS complications in acute malaria: MR findings. AJNR Am J Neuroradiol.

[6] Koch J, Strik WK, Becker T, Flesicher K, Gold R, Hofmann E (1996). Acute organic psychosis after malaria tropica. Nervenarzt.

